# Mr. Anderson's Letter to Mr. Ring, on Vaccination

**Published:** 1807-06

**Authors:** John Ring

**Affiliations:** New Street, Hanover Square


*To the Editors of the Medical and Physical Journal.*

Gentlemen,

The following letter, which I lately received from Dr. Anderson, Physician General, and President of the Medical Board, at Madras, will, I doubt not, afford great satisfaction to your readers.

---

"My Dear Sir,

"I have the pleasure to inform you, that Government, both here and in Bengal, have set on foot a subscription for the benefit of Dr. Jenner, which I sincerely wish may prove productive. I have perused your answer to Mr. Goldson, as it afforded me some entertainment; and think you need not give yourself any trouble about Dr. Rowley, or fifty such opponents.

"*No serious alarm has been caused by the small-pox, in that vast extent of country which is now subject to Great Britain, in India, since the introduction of vaccine inoculation; nor has the vaccine matter, although transferred from one human subject to another for four or five years, produced any other disease.*

"Having found part of your translation of Anstey's Ode to Jenner in the Gentleman's Magazine, I caused it to be inserted in the Madras Gazette; but with regard to the Report of the Medical Council of the Royal Jennerian Society; as there are no Dr. Rowleys in this country, and we have so much evidence in favour of vaccination, I am of opinion, that the publication of it here is unnecessary.

"Be

" Be so good as to thank Dr. Jenner for the copy of " The Evidence at large," which he was so kind to send me; and the Royal Jennerian Society for the honour which they have conferred on me, by electing me a Corresponding Member.

" Sincerely wishing you every felicity, and the full enjoyment of that satisfaction, which is the right of all those, who come forward in the cause of humanity so laudably as you have done,

" I remain, &c.

James Anderson."

At the same time with the foregoing letter, I received from Dr. Anderson a second copy of the last pamphlet which he has thought it necessary to publish on this subject. It is dated 1805, and entitled " Correspondence for the Extermination of the Small-pox." In this publication he informs us, that in consequence of the death of a Rajah from the small-pox, in one of the Northern Circars, the natives are now more sensible of the advantage of vaccination, and more ready to adopt the practice; but notwithstanding the countenance and support of government, beyond what is found in Europe, much still remains to be effected.

In this work Dr. Anderson has republished a letter, dated August the 6th 1784, first inserted in the Government Gazette, in which he says, " I have received from the Right Honourable the Governor, several pamphlets written by Mr. Ring and the Royal Jennerian Society, which it will give me pleasure to transmit to gentlemen desirous of information."—Hence it appears, that Dr. Anderson, as well as the Society which transmitted my publications to India, entertained a more favourable opinion of them than that lately expressed by certain northern reviewers. —These gentlemen had not then illumined the world with their *new light*.

In another letter, dated August the 10th, 1804, also published in the Madras Gazette, Dr. Anderson states, that the cow-pock, under the direction of that Presidency, has resisted the test of fifteen hundred variolous inoculations: and the establishment being so much benefited by the practice, he proposes, that the seventeenth of May, the birth-day of Dr. Jenner, and annual festival of the Royal Jennerian Society, should be marked in their calendar, as an annual festival at Madras.

Dr. Anderson informs us, that independent of other advantages arising from vaccination, it is of no small importance when considered in a pecuniary point of view; since every life which it preserves, in that part of the world, may be valued at eight shillings a year, as a source of revenue.—He also states, that some of the native chiefs countenance vaccination, and even submit their own persons to the practice; and that he has little doubt, the vaccine lancet will soon be as familiar to the Hindoos, as the plough or the shuttle.

From Dr. Anderson's publication we learn, that a reward is held out by the Madras Government, as well to the natives as to our own countrymen, for inoculation; without which it would be idle and foolish to expect any great progress in the extermination of the small-pox. We also learn, that the vaccine matter had in some instances been lost, being taken at too late a period; *in consequence of which, only spurious pustules were produced.*

In the Madras Gazette of April 11, 1805, is a letter from the Government of that Presidency to the Court of Directors, dated February the 22d 1804, of which the following is an extract.

"Vaccine Inoculation introduced."

"Hospital established at the Presidency."

"The introduction of a practice, which holds out such important advantages to the community, will naturally ensure every assistance and encouragement on our part.—We most cordially join in the encomiums which have been passed on your Physician General, Dr. Anderson, for his benevolent and indefatigable exertions in extending this practice; and entirely concur in the measures recommended by the Medical Board, for extending and preserving the benefits of the discovery, in the territories under your superintendance. The co-operation of that Board, with the laudable endeavour of their President, merits our particular approbation; and great praise is due to all the Medical Officers of this Presidency, for their assistance in promoting the practice.

"The establishment and allowances which you have resolved on, for carrying the benevolent purposes of this discovery into effect, have our approbation; and the sentiments expressed in the 126th and 127th paragraphs coincide with our own opinions on the subject."

In the same work is a letter from Mr. Jukes, dated Bushire, March the 13th, 1805, giving an account of his introducing the practice into that part of the Persian dominions.

dominions. This, he informs us, was done by means of equine matter, sent from Vienna; which produced a vesicle similar to the cow-pock, of the most distinct and regular kind.

From the hasty manner in which he travelled through the country, he could not expect to be very successful in diffusing the new inoculation; and he moreover tells us, that they do not know the Persians, who suppose they have any notions of the public good; or that any thing, except pleasure, or money, is capable of engrossing their attention.

Mr. Jukes, however, at first met with some encouragement; and began to indulge the pleasing hope of rapidly diffusing the practice through the whole empire. Numbers of people flocked to his house, in order to be inoculated; and he had already began to circulate pamphlets of instruction in the Persian language, as well as virus; but the jealousy of the Shaikh was awakened, and he was obliged to desist, receiving only the most pointed insult, as his reward. The pretence for rejecting the blessing of vaccination was, that it had sprung from the impure hand of an unbeliever!

This valuable publication of Dr. Anderson concludes with a letter from a native; in which he expresses his grateful acknowledgments for the advantages which he had derived from vaccination, in his own family; and declares, that since the operation, his children are much improved in their health.

Four hundred and twenty-nine thousand, eight hundred and twenty-one, had been successfully vaccinated in the Presidency of Madras, and its dependencies, between the beginning of September, 1802, and the end of May, 1805, at the expence of 55,865 star-pagodas; and two thousand, eight hundred and sixteen of them had been subsequently inoculated for the small-pox, which they all resisted.—This is a sufficient refutation of all the calumnies and falsehoods, which are daily circulated with so much industry, and effrontery, here at home.

I am, &c.

JOHN RING.

*New Street, Hanover Square, Apr. 27, 1807.*

General Abstract of Persons Vaccinated at the Presidency of Madras, and at the Subordinate Stations subject to the Authority of this Government, from the 1st of September, 1805, to the 31st of August, 1806.

| Divisions. | Medical Gentlemen superintending Vaccine Inoculation. | Stations. | Cast and Sex of Patients duly Vaccinated. Christians | | Hindoos. | | Mahometans. | | Total Vaccinated. | | Grand Total. |
|---|---|---|---|---|---|---|---|---|---|---|---|
| | | | Males | Females | Males | Females | Males | Females | Males | Females | |
| Northern. | Surgeon Owen - - - - - | Ganjam district - - | 8 | 6 | 2054 | 1917 | 9 | 5 | 2171 | 1928 | 4099 |
| | Surgeon Baillie - - - - | Vizagapatam district - | 87 | 124 | 1647 | 1377 | 143 | 150 | 1877 | 1651 | 3528 |
| | Surgeon Norman - - - | Ingeram district - - | 37 | 30 | 582 | 387 | 29 | 28 | 648 | 455 | 1103 |
| | Surgeon Jameson - - - | Rajahmundry district - | 236 | 178 | 3418 | 2416 | 49 | 18 | 3698 | 2612 | 6310 |
| | Surgeon Street - - - - | Masulipatam district - | 290 | 255 | 1541 | 1373 | 79 | 60 | 1910 | 1688 | 3598 |
| Centre. | Surgeon Thompson - - - | Guntoor district - - | | | 3783 | 3320 | 144 | 93 | 3927 | 3413 | 7340 |
| | Surgeon Prichard - - - | Vellore district - - - | 170 | 178 | 211 | 190 | 206 | 219 | 587 | 587 | 1174 |
| | Assistant Surgeon Scott - | Nellore district - - - | 293 | 234 | 2329 | 2293 | 308 | 143 | 2930 | 2670 | 5600 |
| | Surgeon M'Kenzie - - - | Presidency and Jahgier | 3460 | 2662 | 10636 | 7663 | 2368 | 1411 | 16464 | 11734 | 28198 |
| | Assistant Surgeon Campbell | Trepasore district - - | 297 | 192 | 347 | 319 | 71 | 52 | 615 | 563 | 1178 |
| | Surgeon Dalton - - - - | Arnee district - - - | 640 | 423 | 2199 | 1581 | 705 | 421 | 3544 | 2425 | 5969 |
| | Surgeon Duncan - - - | Cuddalore district - - | 401 | 355 | 628 | 624 | 111 | 74 | 1140 | 1043 | 2183 |
| | Surgeon Tozer - - - - | Ryacottah district - - | 62 | 104 | 714 | 905 | 19 | 22 | 807 | 1019 | 1826 |
| | Surgeon Mudie - - - - | Salem district - - - | 763 | 782 | 2428 | 2434 | 31 | 29 | 3222 | 3245 | 6467 |

| | | | | | | | | | | | |
|---|---|---|---|---|---|---|---|---|---|---|---|
| SOUTHERN. | Assistant Surgeon Boodle - | Ramnadporam district - | 378 | 312 | 6822 | 4948 | 223 | 157 | 7413 | 5387 | 12800 |
| | Assistant Surgeon M'Cabe | Palamcottah district - | 144 | 101 | 519 | 252 | 2 | 2 | 665 | 455 | 1120 |
| | Assistant Surgeon Christy | Madura district - - | 398 | 336 | 2573 | 2091 | 155 | 87 | 3126 | 2514 | 5640 |
| | Assistant Surgeon Alexander | Dindigul district - - | 926 | 715 | 3771 | 3071 | 201 | 158 | 4898 | 3944 | 8842 |
| | Surgeon Spalding - - - | Negapatam district - - | 136 | 142 | 1003 | 1056 | 12 | 8 | 1151 | 1206 | 2357 |
| | Surgeon James - - - - | Tanjore district - - - | 1339 | 1139 | 2441 | 2157 | 45 | 17 | 3825 | 3313 | 7138 |
| | Surgeon Hay - - : - - | Trichinopoly district - | 325 | 406 | 1953 | 2018 | 150 | 155 | 2428 | 2579 | 5007 |
| | Staff Surgeon Harris - - | Quilon District - - - | 1129 | 1093 | 2405 | 1890 | 329 | 154 | 3863 | 3137 | 7000 |
| CEDED. | Surgeon Peyton - - - - | Bellary district - - - | 70 | 79 | 518 | 604 | 147 | 142 | 735 | 825 | 1560 |
| | Assistant Surgeon Wyse - | Cumbum district - - | 2 | | 611 | 507 | 82 | 49 | 695 | 556 | 1251 |
| MYSORE. | Surgeon Scarman - - - | Seringapatam district - | 547 | 498 | 831 | 830 | 461 | 446 | 8839 | 1774 | 3613 |
| | Missioner Dubois - - - | Mysore district - - - | 68 | 65 | 3651 | 3496 | 211 | 162 | 3930 | 3723 | 7653 |
| | Assistant Surgeon Heyne - | Bangalore district - - | 3 | 1 | 2146 | 1653 | 347 | 316 | 2496 | 1970 | 4466 |
| MALABAR. | Surgeon White - - - - | Cannanore district - - | 47 | 46 | 171 | 67 | 209 | 29 | 427 | 142 | 569 |
| | Assistant Surgeon Palmer - | Kitcuddy district - - | 138 | 21 | 5745 | 2306 | 3035 | 1075 | 8918 | 3402 | 12320 |
| | Assistant Surgeon Napier - | Mangalore district - - | 844 | 563 | 1762 | 981 | 136 | 63 | 2742 | 1607 | 4349 |
| | Surgeon Meek - - - - | Calicut district - - - | | | 4487 | 2508 | 1582 | 743 | 6069 | 3251 | 9320 |
| | Surgeon Morgan - - - - | Tellicherry district - - | 179 | 101 | 2399 | 1296 | 424 | 97 | 3002 | 1494 | 4496 |
| | | | | | | | | | 101762 | 76312 | 178074 |

*Fort St. George, Sept. 1, 1806.*

A. M'KENZIE, M. D. *Superintendant General of Vaccine Inoculation.*

